# 2,9-Diiodo­hexa­cyclo­[9.3.1.1^2,6^.1^4,8^.1^9,13^.0^1,8^]octa­deca­ne

**DOI:** 10.1107/S1600536812026797

**Published:** 2012-07-04

**Authors:** Savvas Ioannou, Eleni Moushi

**Affiliations:** a Chemistry Department, University of Cyprus, Nicosia 1678, Cyprus

## Abstract

The title compound, C_18_H_24_I_2_, has an adamantanoid structure with tetra­hedral cages having four C atoms lying on the same plane [(I—)C—C—C—C(—I) torsion angle = 0°]. The plane is extended by the two I atoms, each having a deviation of 1.0 (6) Å [C—C—C—I torsion angle = 178.9 (4)°]. The central C—C bond connecting the two quaternary carbons seems enlarged [1.593 (9) Å] in comparison to the corresponding bond in [2]diadamantane [1.554 (3) Å]. This is attributed to the presence of the electronegative I atoms, which affect inductively the C atoms of the four-C-atom plane, making the central C—C bond weaker.

## Related literature

For reviews on noradamantene and analogous pyramidalized alkenes, see: Borden (1989 ▶, 1996 ▶); Vázquez & Camps (2005 ▶). For the synthesis of the precursor, hepta­cyclo­[9.3.1.1^2,6^.1^4,8^.1^9,13^.0^1,9^.0^2,8^]octa­decane, see: Ioannou & Nicolaides (2009 ▶); Renzoni *et al.* (1986 ▶) and for the synthesis of [2]diadamantane, see: McKervey (1980 ▶); Graham *et al.* (1973 ▶). For related reactions on diadamantane systems, see: Sosnowski *et al.* (1984 ▶). For the use of iodine as a trapping agent for the inter­mediate radicals of a reaction, see: Castello (1984 ▶); Wojnarovits & Laverne (1996 ▶). For iodine as a catalyst, see: Mullineaux & Raley (1963 ▶); Slaugh *et al.* (1963 ▶).
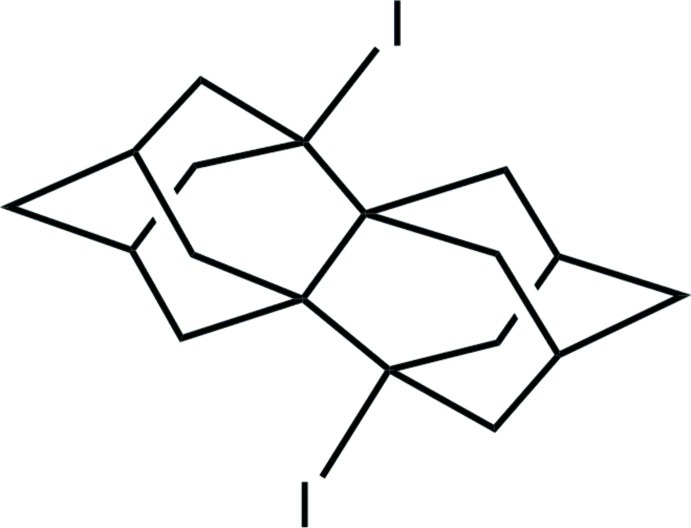


## Experimental

### 

#### Crystal data

C_18_H_24_I_2_
*M* *_r_* = 494.17
Triclinic, 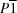

*a* = 6.8912 (8) Å
*b* = 6.9725 (9) Å
*c* = 8.9927 (10) Å
α = 67.964 (11)°
β = 74.368 (10)°
γ = 78.258 (10)°
*V* = 383.16 (9) Å^3^
*Z* = 1
Mo *K*α radiation
μ = 4.09 mm^−1^
*T* = 100 K
0.18 × 0.05 × 0.03 mm

#### Data collection

Oxford Diffraction SuperNova Dual (Cu at 0) Atlas diffractometer
Absorption correction: multi-scan (*CrysAlis RED*; Oxford Diffraction, 2008 ▶) *T*_min_ = 0.527, *T*_max_ = 1.000
2333 measured reflections
1346 independent reflections
1284 reflections with *I* > 2σ(*I*)
*R*_int_ = 0.040

#### Refinement

*R*[*F* ^2^ > 2σ(*F* ^2^)] = 0.032
*wR*(*F* ^2^) = 0.085
*S* = 1.10
1346 reflections
91 parameters
18 restraints
H-atom parameters constrained
Δρ_max_ = 1.50 e Å^−3^
Δρ_min_ = −0.62 e Å^−3^

### 

Data collection: *CrysAlis CCD* (Oxford Diffraction, 2008 ▶ ▶); cell refinement: *CrysAlis CCD*; data reduction: *CrysAlis RED* (Oxford Diffraction, 2008 ▶ ▶); program(s) used to solve structure: *SHELXS97* (Sheldrick, 2008 ▶ ▶); program(s) used to refine structure: *SHELXL97* (Sheldrick, 2008 ▶ ▶); molecular graphics: *DIAMOND* (Brandenburg, 2006 ▶ ▶) and *Mercury* (Macrae *et al.*, 2006 ▶ ▶); software used to prepare material for publication: *WinGX* (Farrugia, 1999 ▶ ▶) and *publCIF* (Westrip, 2010 ▶ ▶).

## Supplementary Material

Crystal structure: contains datablock(s) I, global. DOI: 10.1107/S1600536812026797/zj2079sup1.cif

Structure factors: contains datablock(s) I. DOI: 10.1107/S1600536812026797/zj2079Isup2.hkl

Supplementary material file. DOI: 10.1107/S1600536812026797/zj2079Isup3.cdx

Supplementary material file. DOI: 10.1107/S1600536812026797/zj2079Isup4.cml

Additional supplementary materials:  crystallographic information; 3D view; checkCIF report

## Figures and Tables

**Fig. 1.**
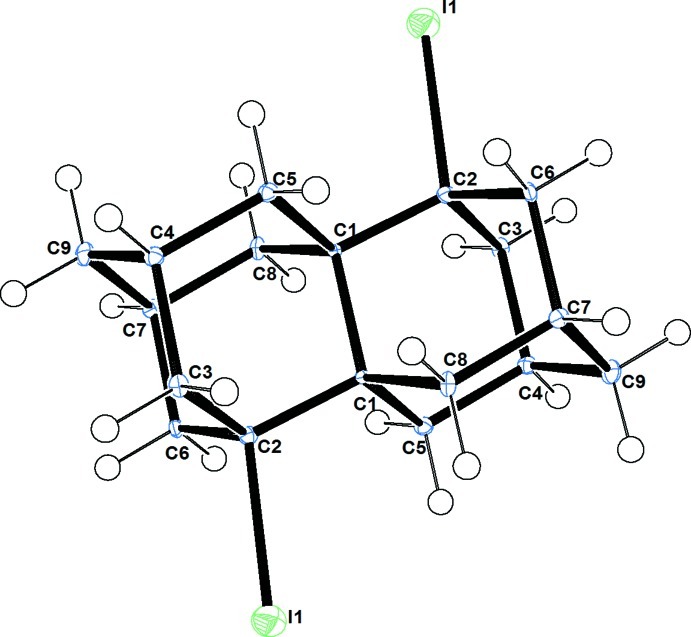
Structure of the title compound 2,9-Diiodo-hexacyclo [9.3.1.12,6.14,8.19,13.01,8] octadecane with the atom-labelling. Displacement elipsoids are drawn at the 50% probability level.

**Fig. 2.**
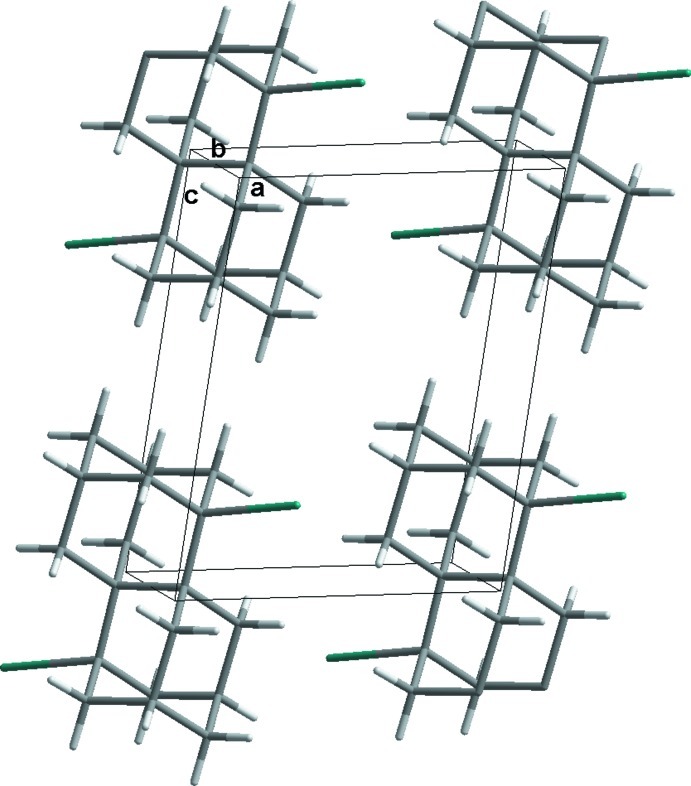
Molecular packing of the title compound, viewed along [0 1 0].

**Fig. 3.**
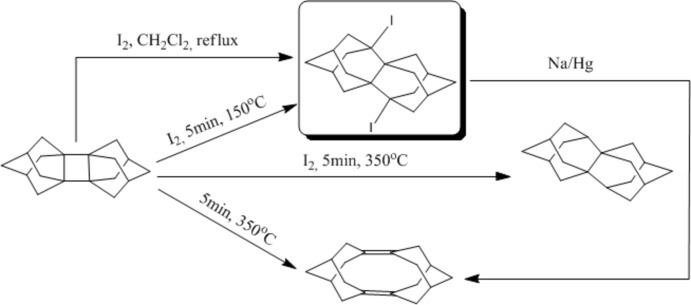
Synthesis of the title compound.

## supplementary crystallographic information

### Comment

Adamantanoids are cage structures having adamantane as a repeated unit (McKervey 1980\). Their nomenclature derives from the number of the common carbon atoms connecting every adamantane unit to each other (Graham *et al.* 1973\). 2,9-Diiodo [2]diadamantane (title compound) was the main product of thermolysis of heptacyclo [9.3.1.1^2,6^.1^4,8^.1^9,13^.0^1,9^.0^2,8^] octadecane in the presence of iodine at 150°C. Iodine was used as a trapping agent (Castello 1984\, Wojnarovits *et al.* 1996\) for the intermediate radicals of the reaction. The corresponding iodides helped in the understanding of the reaction mechanism. Iodine was applied initially at the reaction conditions (5 min, 350°C) predefined for the synthesis of pentacyclo [9.3.1.1^2,6^.1^4^,8.1^9,13^] octadeca-di-1(2),8(9)-ene but it acted as a catalyst instead (Slaugh *et al.* 1963\, Mullineaux *et al.* 1963\), leading the reaction spontaneously to the more favored thermodynamically product [2]diadamantane (figure 3). At lower temperature (150°C) the title compound was isolated as the main product of the reaction among other minor products. Another method of producing 2,9-diiodo[2]diadamantane quantitatively is by refluxing the starting material in dichloromethane with 2 equivalents of iodine (lower temperature). Other solvents were used as well, like carbon tetrachloride and chloroform but the reaction was slower having lower yields. Dichloromethane was the most suitable solvent probably due to its bigger dipole moment that helps the homolysis. The title compound has its own interest as the first substituted [2]diadamantane at the specific positions considered by others as the more difficult positions to functionalize (Sosnowski *et al.* 1984\).

### Experimental

Synthesis of 2,9-diiodo-hexacyclo[9.3.1.1^2,6^.1^4,8^.1^9,13^.0^1,8^]octadecane. Heptacyclo[9.3.1.1^2,6^.1^4,8^.1^9,13^.0^1,9^.0^2,8^]octadecane (68 mg, 0.28 mmol), iodine (131 mg, 0.52 mmol) and dichloromethane (10 ml) were refluxed in a round bottom flask for 5 h. Another 10 ml of dichloromethane were added when the mixture cooled down and extracted with 1x30 ml saturated aqueous sodium thiosulfate for the removal of the iodine excess. The organic phase was then dried with anhydrous Na_2_SO_4_ and removed under vacuum to give 86 mg (62%) of a white solid (title compound) that was recrystallized (hexane/dichloromethane 5:1) to give pure colorless crystals(mp 240–242°C).

### Refinement

The H atoms are positioned with idealized geometry and refined using a riding model with *U*_iso_(H) = 1.2 of *U*_eq_ (C).

### Crystal data

**Table:**

C_18_H_24_I_2_	*Z* = 1
*M**_r_* = 494.17	*F*(000) = 238
Triclinic, *P*1	*D*_x_ = 2.141 Mg m^−^^3^
*a* = 6.8912 (8) Å	Mo *K*α radiation, λ = 0.71073 Å
*b* = 6.9725 (9) Å	Cell parameters from 1750 reflections
*c* = 8.9927 (10) Å	θ = 3.1–28.8°
α = 67.964 (11)°	µ = 4.09 mm^−^^1^
β = 74.368 (10)°	*T* = 100 K
γ = 78.258 (10)°	Polyhedral, colorless
*V* = 383.16 (9) Å^3^	0.18 × 0.05 × 0.03 mm

### Data collection

**Table:**

Oxford Diffraction SuperNova Dual (Cu at 0) Atlas diffractometer	1346 independent reflections
Radiation source: SuperNova (Mo) X-ray Source	1284 reflections with *I* > 2σ(*I*)
Mirror monochromator	*R*_int_ = 0.040
Detector resolution: 10.4223 pixels mm^-1^	θ_max_ = 25.0°, θ_min_ = 3.1°
ω scans	*h* = −8→8
Absorption correction: multi-scan (*CrysAlis RED*; Oxford Diffraction, 2008\)	*k* = −8→8
*T*_min_ = 0.527, *T*_max_ = 1.000	*l* = −10→10
2333 measured reflections	

### Refinement

**Table:**

Refinement on *F*^2^	Primary atom site location: structure-invariant direct methods
Least-squares matrix: full	Secondary atom site location: difference Fourier map
*R*[*F*^2^ > 2σ(*F*^2^)] = 0.032	Hydrogen site location: inferred from neighbouring sites
*wR*(*F*^2^) = 0.085	H-atom parameters constrained
*S* = 1.10	*w* = 1/[σ^2^(*F*_o_^2^) + (0.0358*P*)^2^ + 1.7753*P*] where *P* = (*F*_o_^2^ + 2*F*_c_^2^)/3
1346 reflections	(Δ/σ)_max_ < 0.001
91 parameters	Δρ_max_ = 1.50 e Å^−^^3^
18 restraints	Δρ_min_ = −0.62 e Å^−^^3^

### Special details

**Table:**

Geometry. All e.s.d.'s (except the e.s.d. in the dihedral angle between two l.s. planes) are estimated using the full covariance matrix. The cell e.s.d.'s are taken into account individually in the estimation of e.s.d.'s in distances, angles and torsion angles; correlations between e.s.d.'s in cell parameters are only used when they are defined by crystal symmetry. An approximate (isotropic) treatment of cell e.s.d.'s is used for estimating e.s.d.'s involving l.s. planes.
Refinement. Refinement of *F*^2^ against ALL reflections. The weighted *R*-factor *wR* and goodness of fit *S* are based on *F*^2^, conventional *R*-factors *R* are based on *F*, with *F* set to zero for negative *F*^2^. The threshold expression of *F*^2^ > σ(*F*^2^) is used only for calculating *R*-factors(gt) *etc*. and is not relevant to the choice of reflections for refinement. *R*-factors based on *F*^2^ are statistically about twice as large as those based on *F*, and *R*- factors based on ALL data will be even larger.

### Fractional atomic coordinates and isotropic or equivalent isotropic displacement parameters (Å^2^)

**Table:**

	*x*	*y*	*z*	*U*_iso_*/*U*_eq_	
I1	0.33440 (5)	0.02562 (5)	0.21656 (4)	0.01697 (17)	
C1	0.0899 (7)	0.4075 (7)	0.0021 (6)	0.0031 (10)	
C2	0.0901 (8)	0.2866 (8)	0.1856 (6)	0.0050 (10)	
C3	−0.1085 (8)	0.1932 (7)	0.2800 (6)	0.0050 (10)	
H3A	−0.1001	0.1131	0.3930	0.006*	
H3B	−0.1335	0.1009	0.2306	0.006*	
C4	−0.2820 (8)	0.3723 (8)	0.2741 (6)	0.0055 (10)	
H4	−0.4107	0.3142	0.3315	0.007*	
C5	−0.2919 (8)	0.5004 (8)	0.0951 (6)	0.0054 (10)	
H5A	−0.4017	0.6128	0.0921	0.006*	
H5B	−0.3203	0.4124	0.0436	0.006*	
C6	0.1293 (8)	0.4238 (8)	0.2722 (6)	0.0056 (10)	
H6A	0.1357	0.3414	0.3853	0.007*	
H6B	0.2576	0.4803	0.2173	0.007*	
C7	−0.0445 (8)	0.6011 (8)	0.2666 (6)	0.0053 (10)	
H7	−0.0197	0.6915	0.3191	0.006*	
C8	−0.0557 (8)	0.7290 (8)	0.0884 (6)	0.0052 (10)	
H8A	0.0695	0.7912	0.0319	0.006*	
H8B	−0.1661	0.8408	0.0861	0.006*	
C9	−0.2454 (8)	0.5112 (8)	0.3588 (6)	0.0072 (10)	
H9A	−0.2393	0.4303	0.4722	0.009*	
H9B	−0.3554	0.6230	0.3576	0.009*	

### Atomic displacement parameters (Å^2^)

**Table:**

	*U*^11^	*U*^22^	*U*^33^	*U*^12^	*U*^13^	*U*^23^
I1	0.0167 (2)	0.0151 (2)	0.0163 (3)	0.00018 (16)	−0.00330 (17)	−0.00354 (17)
C1	0.0030 (13)	0.0030 (13)	0.0038 (13)	−0.0003 (9)	−0.0010 (9)	−0.0017 (9)
C2	0.005 (2)	0.006 (2)	0.006 (2)	0.0000 (19)	−0.002 (2)	−0.003 (2)
C3	0.008 (2)	0.002 (2)	0.005 (2)	−0.0021 (19)	−0.001 (2)	−0.0014 (19)
C4	0.0049 (13)	0.0056 (13)	0.0058 (13)	−0.0009 (9)	−0.0009 (9)	−0.0017 (9)
C5	0.0045 (13)	0.0057 (13)	0.0057 (13)	−0.0008 (9)	−0.0008 (9)	−0.0016 (9)
C6	0.005 (2)	0.009 (2)	0.003 (2)	−0.001 (2)	−0.001 (2)	−0.002 (2)
C7	0.005 (2)	0.006 (2)	0.007 (3)	−0.001 (2)	−0.002 (2)	−0.004 (2)
C8	0.007 (2)	0.006 (2)	0.004 (2)	−0.003 (2)	0.000 (2)	−0.002 (2)
C9	0.007 (2)	0.008 (2)	0.005 (3)	−0.002 (2)	0.001 (2)	−0.001 (2)

### Geometric parameters (Å, º)

**Table:**

I1—C2	2.202 (5)	C5—H5A	0.9700
C1—C2	1.545 (7)	C5—H5B	0.9700
C1—C8^i^	1.549 (6)	C6—C7	1.532 (7)
C1—C5^i^	1.550 (7)	C6—H6A	0.9700
C1—C1^i^	1.593 (9)	C6—H6B	0.9700
C2—C3	1.529 (7)	C7—C8	1.528 (7)
C2—C6	1.541 (7)	C7—C9	1.530 (7)
C3—C4	1.539 (7)	C7—H7	0.9800
C3—H3A	0.9700	C8—C1^i^	1.549 (6)
C3—H3B	0.9700	C8—H8A	0.9700
C4—C9	1.529 (7)	C8—H8B	0.9700
C4—C5	1.532 (7)	C9—H9A	0.9700
C4—H4	0.9800	C9—H9B	0.9700
C5—C1^i^	1.550 (7)		
			
C2—C1—C8^i^	113.1 (4)	C4—C5—H5B	109.4
C2—C1—C5^i^	112.8 (4)	C1^i^—C5—H5B	109.4
C8^i^—C1—C5^i^	106.0 (4)	H5A—C5—H5B	108.0
C2—C1—C1^i^	106.0 (5)	C7—C6—C2	108.3 (4)
C8^i^—C1—C1^i^	109.7 (5)	C7—C6—H6A	110.0
C5^i^—C1—C1^i^	109.2 (5)	C2—C6—H6A	110.0
C3—C2—C6	108.4 (4)	C7—C6—H6B	110.0
C3—C2—C1	112.5 (4)	C2—C6—H6B	110.0
C6—C2—C1	112.2 (4)	H6A—C6—H6B	108.4
C3—C2—I1	106.6 (3)	C8—C7—C9	109.7 (4)
C6—C2—I1	105.3 (3)	C8—C7—C6	109.9 (4)
C1—C2—I1	111.4 (3)	C9—C7—C6	109.8 (4)
C2—C3—C4	108.4 (4)	C8—C7—H7	109.1
C2—C3—H3A	110.0	C9—C7—H7	109.1
C4—C3—H3A	110.0	C6—C7—H7	109.1
C2—C3—H3B	110.0	C7—C8—C1^i^	111.4 (4)
C4—C3—H3B	110.0	C7—C8—H8A	109.3
H3A—C3—H3B	108.4	C1^i^—C8—H8A	109.3
C9—C4—C5	109.9 (4)	C7—C8—H8B	109.3
C9—C4—C3	109.5 (4)	C1^i^—C8—H8B	109.3
C5—C4—C3	109.7 (4)	H8A—C8—H8B	108.0
C9—C4—H4	109.2	C4—C9—C7	108.3 (4)
C5—C4—H4	109.2	C4—C9—H9A	110.0
C3—C4—H4	109.2	C7—C9—H9A	110.0
C4—C5—C1^i^	111.3 (4)	C4—C9—H9B	110.0
C4—C5—H5A	109.4	C7—C9—H9B	110.0
C1^i^—C5—H5A	109.4	H9A—C9—H9B	108.4
			
C8^i^—C1—C2—C3	−58.8 (5)	C9—C4—C5—C1^i^	61.2 (5)
C5^i^—C1—C2—C3	−179.0 (4)	C3—C4—C5—C1^i^	−59.2 (5)
C1^i^—C1—C2—C3	61.4 (6)	C3—C2—C6—C7	−62.1 (5)
C8^i^—C1—C2—C6	178.6 (4)	C1—C2—C6—C7	62.7 (5)
C5^i^—C1—C2—C6	58.4 (5)	I1—C2—C6—C7	−175.9 (3)
C1^i^—C1—C2—C6	−61.1 (6)	C2—C6—C7—C8	−59.0 (5)
C8^i^—C1—C2—I1	60.8 (5)	C2—C6—C7—C9	61.7 (5)
C5^i^—C1—C2—I1	−59.4 (4)	C9—C7—C8—C1^i^	−61.8 (5)
C1^i^—C1—C2—I1	−178.9 (4)	C6—C7—C8—C1^i^	59.0 (5)
C6—C2—C3—C4	62.2 (5)	C5—C4—C9—C7	−59.8 (5)
C1—C2—C3—C4	−62.4 (5)	C3—C4—C9—C7	60.8 (5)
I1—C2—C3—C4	175.2 (3)	C8—C7—C9—C4	60.0 (5)
C2—C3—C4—C9	−62.1 (5)	C6—C7—C9—C4	−60.9 (5)
C2—C3—C4—C5	58.6 (5)		

Symmetry code: (i) −*x*, −*y*+1, −*z*.
